# In silico validation of a new classifier, PCSCG_ier_, for predicting recurrence‐free survival in prostate cancer patients: *Evidence from multiple datasets*

**DOI:** 10.1002/ctm2.1105

**Published:** 2023-01-15

**Authors:** Meng Zhang, Zichen Bian, Jia Chen, Lei Chen, Jun Zhou, Qingsong Niu, Zongyao Hao, Jialin Meng, Chaozhao Liang

**Affiliations:** ^1^ Department of Urology The First Affiliated Hospital of Anhui Medical University Hefei China; ^2^ Institute of Urology Anhui Medical University Hefei China; ^3^ Anhui Province Key Laboratory of Genitourinary Diseases Anhui Medical University Hefei China; ^4^ Institute of Urology of Shenzhen University The Third Affiliated Hospital of Shenzhen University Shenzhen Luohu Hospital Group Shenzhen China

Dear Editor,

Prostate cancer (PCa) is forecasted to be second death‐related cancer worldwide.^1^ PCa stem cells (PCSCs) are slow‐cycling cells that participate in the carcinogenesis, progression and therapeutic resistance of PCa. Here, we correlated PCSCs‐related genes (PCSCGs) with PCa patients’ recurrence‐free survival (RFS) and established a PCSC‐related gene‐based classifier (termed PCSCG_ier_). The clinicopathological features of recruited cohorts were listed in Table S2. The shRNA primer sequence and antibody sources used in the study are listed in Tables S3 and S4. The methods details were demonstrated in **Online Materials and Methods**.

Gene chip profile of the C4‐2 sphere formation group and parental cells identified 101 up‐regulated and 26 down‐regulated differentially expressed PCSCGs (Figure 1A and Figure S1A,B), and 33 of them were found to be RFS‐correlated in the TCGA‐PRAD dataset (Figure 1B and Table S1). LASSO Cox regression was performed to select candidates and establish the prognostic classifier (Figure 1C,D). *FAM83D, FAM129A, CDC20, GINS2, FJX1* and *C16orf59* were ultimately included to construct the signature, and risk score was defined as sum of these gene expression levels multiplying their regression coefficients (Table S1). For the six PCSCGs enrolled in the classifier, five of them were higher expressed in the stem cell enriched C4‐2 cell group, and the higher expression of these genes were correlated with unfavorable prognosis of PCa patients (hazard ratio (HR) > 1, log‐rank *p*‐value < .05). Besides, we found that FAM129A was lower expressed in the stem cell enriched C4‐2 cell group, and the lower expression of this gene was correlated with unfavorable prognosis of PCa patients (HR < 1, log‐rank *p*‐value < .05). Subsequently, patients in TCGA‐PRAD dataset were allocated to low‐risk subgroup (STME‐L) and high‐risk subgroup (STME‐H) based on the median risk score.

**FIGURE 1:**
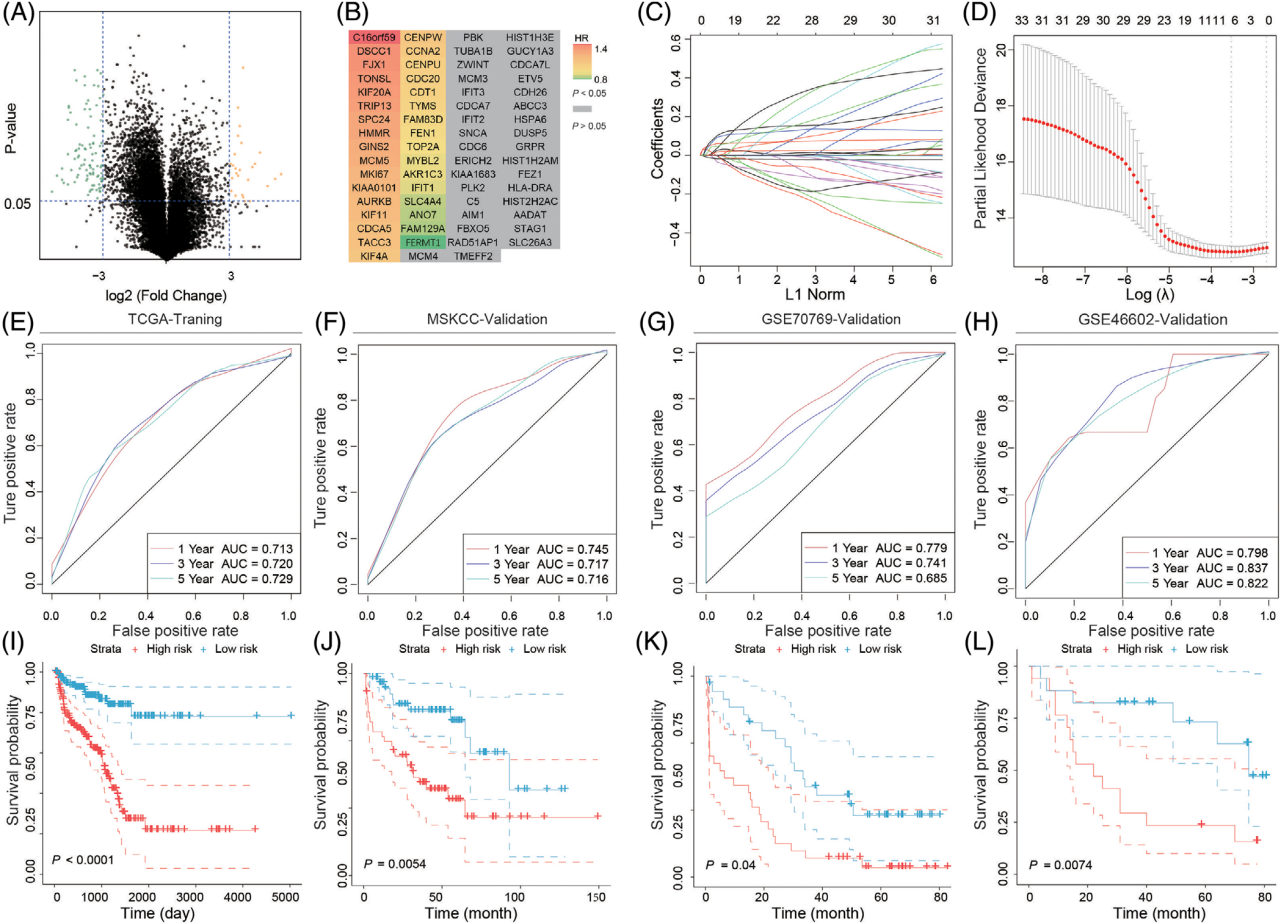
The identification of prostate cancer stem cell‐related genes (PCSCGs) and the establishment and multiple datasets validation of recurrence‐free survival (RFS) indicators based on the PCSCGs. (**A**) Volcano plot showing the differentially expressed genes (DEGs) between the prostate cancer stem cell‐enriched C4‐2 group and the regular C4‐2 group, *p*‐value was calculated by t‐test. (**B**) Heatmap displaying the expression difference between the prostate cancer stem cell‐enriched C4‐2 group and the regular C4‐2 group, *p*‐value was calculated by Cox regression analysis. (**C**) LASSO coefficient profiles of the PCSCGs associated with RFS of prostate cancer based on TCGA‐PRAD cohort. (**D**) Plots of the cross‐validation error rates. (**E–I**) Performance of PCSCG_ier_ in the TCGA‐PRAD training set. (**F–L**) External validation of PCSCG_ier_ in the Memorial Sloan Kettering Cancer Center (MSKCC), GSE70769 and GSE46602 datasets. (**E–H**) ROC curves showing the predictive value of PCSCG_ier_. (**I–L**) Kaplan–Meier plots and log‐rank analyses showing the survival difference between the high risk subgroup (STEM‐H) and low risk subgroup (STEM‐L). *p*‐Value for Kaplan–Meier survival plot was calculated by log‐rank analysis.

Predictive values of this classifier were suggested by the ROC curve analysis, which had a time‐dependent area under the curve of .713 at 1 year, .720 at 3 years and .729 at 5 years (Figure 1E). Kaplan–Meier curves and log‐rank analysis suggested that STME‐H patients mostly had poorer survival outcomes than patients belonged to STME‐L (log‐rank *p* < .0001, Figure 1I). We validated the predictive value of PCSCG_ier_ in the MSKCC, GSE70769 and GSE46602 datasets and all results affirmed the application value and stability of PCSCG_ier_ (Figure 1F–L). We compared the predictive value of PCSCG_ier_ with the T stage, PSA level and Gleason score and performed nomogram analysis by combining all available critical features, and findings suggested that our classifier added predictive value to those features (Figure S2). The subgroup analyses suggested the signature was still applicable in different clinicopathological subsets (Figures S2 and S3).

We then found that high‐risk patients mostly had higher Gleason scores and higher tumour stages. They mostly belonged to immune‐exhausted and nonimmune subgroups,^2^ to the LumB subgroup^3^ and to the C3 and C4 subgroups^4^ (Figure 2A,B). Previously, we found that PCa patients less than 60‐year‐old with an activated immune response mostly had favorable survival outcomes, but we observed that patients with a higher PCSCG_ier_ score accompanied by an activated immune microenvironment had the worst survival outcomes (Figure 2C). In addition, STME‐H patients who also belonged to the C3 subgroup were found to have the most unfavorable prognosis (Figure 2D).

**FIGURE 2:**
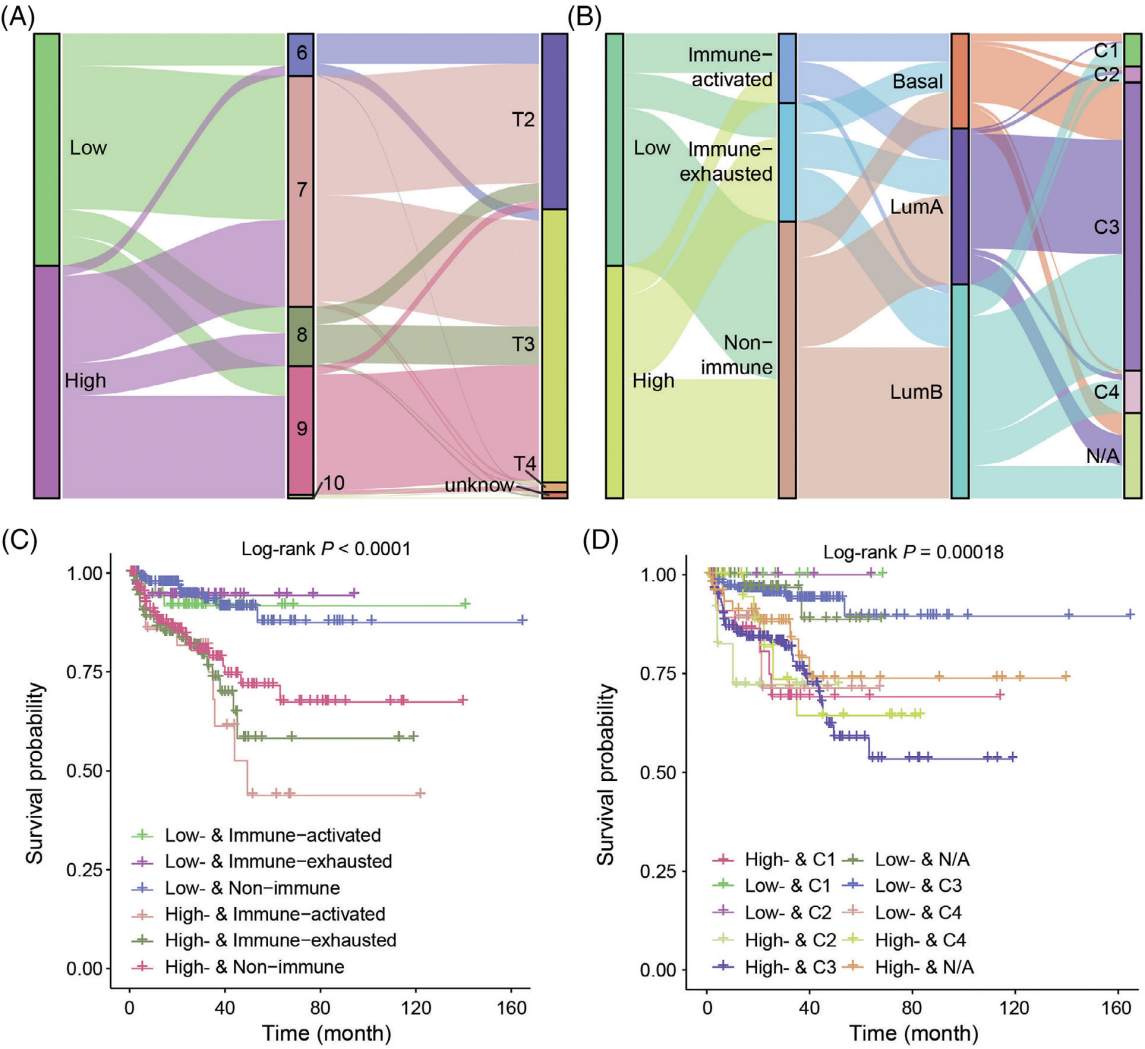
Accurate stratification of prostate cancer patients with different outcomes. (**A** and **B**) Visualizing the correlation between the STEM‐H and STEM‐L of recurrence, determined by the prostate cancer stem cell‐related gene‐based classifier (PCSCG_ier_), and certain clinicopathological features and previously established classifiers. (**C**) Kaplan–Meier plot and log‐rank analysis comparing the survival outcomes within subgroups determined by PCSCG_ier_ and immune activated status, *p‐*value was calculated by log‐rank test. (**D**) Kaplan–Meier plot and log‐rank analysis comparing the survival outcomes within subgroups determined by PCSCG_ier_ and Thorsson et al.’s six immune subtypes, *p‐*value was calculated by log‐rank test.

We explored the differences between STEM‐H and STEM‐L in the TCGA‐PRAD dataset. The results indicated that the DNA repair pathways were activated in the STEM‐H (all *p* < .05, Figure 3A). Therefore, we evaluated the genetic alterations among the STEM‐H and STEM‐L, and the amplifications and deletions at both the arm level and the focal level were more common in the STEM‐H (all *p* < .05, Figure 3B,C). We found that STEM‐H patients had a higher tumour mutation burden (*p* < .001, Figure 3D). We further investigated the correlation between gene mutation and PCSCGier, and observed that *TP53* and *SPOP* mutations were significantly correlated with the scores of PCSCGier (*p* < .05, Figure 3E). GSVA of 50 hallmark classical pathways showed that there was high activation of E2F targets, epithelial–mesenchymal transition, and several immune activation‐associated pathways in the STEM‐H patients, while the STEM‐L patients showed activation of androgen and estrogen responses, PI3K/AKT/MTOR signalling and adipogenesis pathways (Figure 3F). We observed that the STEM‐H patients were more sensitive to treatment with the AKT inhibitors VII, lapatinib and rapamycin (Figure 3G). The STEM‐L patients were more sensitive to treatment with AUY922, CGP‐082996, AZ628, CHIR‐99021, BAY 61–3606 and cyclopamine (Figure 3H). The other 35 potentially beneficial drugs are listed in Figure S4.

**FIGURE 3:**
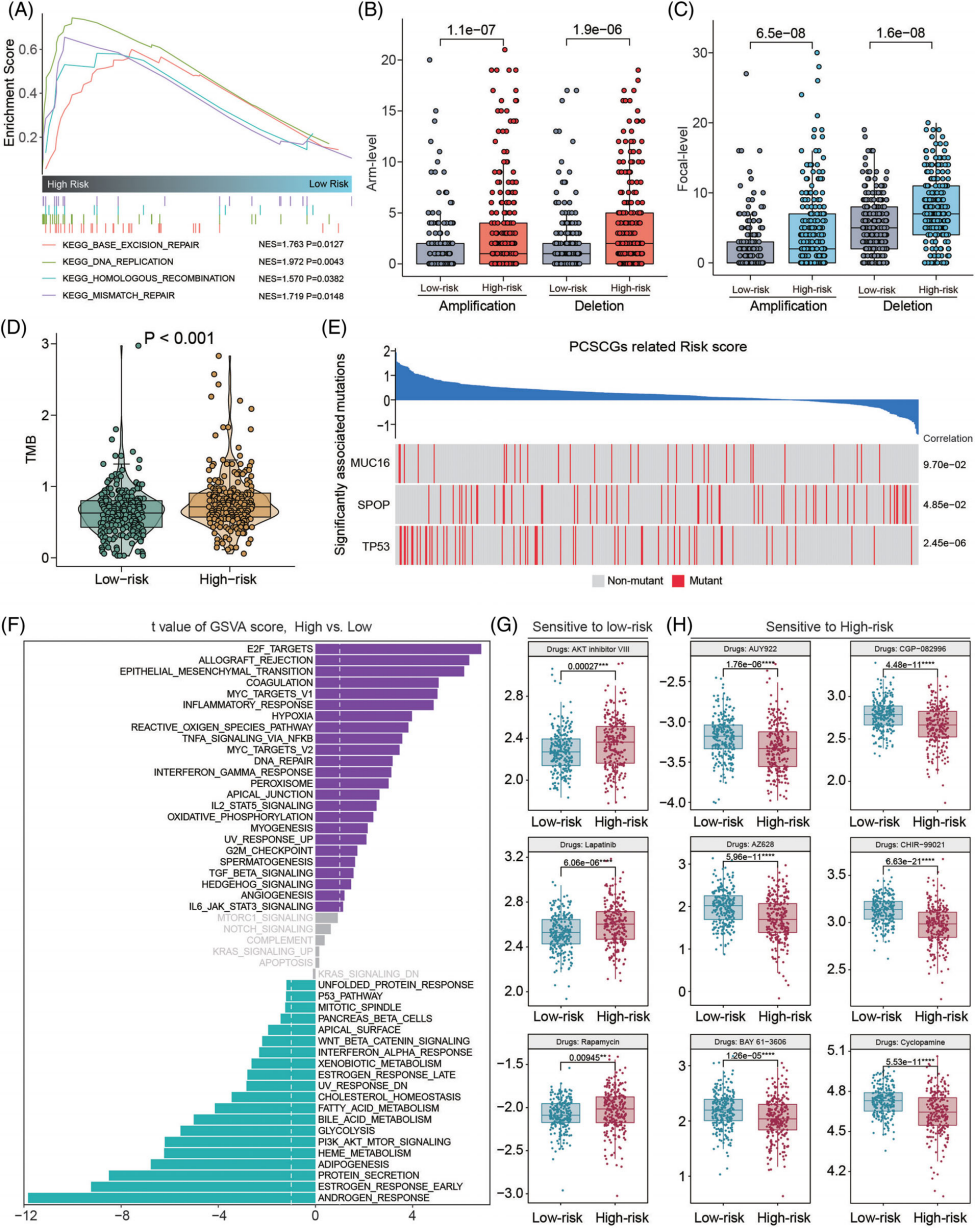
Systematic screening of effective inhibitors for prostate cancer patients with different risks of recurrence. (**A**) gene set enrichment analysis (GSEA) identified the differences between STEM‐H and STEM‐L subgroups at the pathway level. (**B**) High‐risk prostate cancer patients had more arm‐level amplifications and deletions than low‐risk patients; *p‐*value was calculated by Student's *t*‐test. (**C**) High‐risk prostate cancer patients had higher focal levels of amplification and deletion than low‐risk patients; *p‐*value was calculated by Student's *t*‐test. (**D**) High‐risk prostate cancer patients had a higher tumour mutation burden (TMB) than low‐risk patients; *p‐*value was calculated by Student's *t*‐test. (**E**) Correlations between *MUC16*, *SPOP* and *TP53* mutation and risk score generated by the prostate cancer stem cell‐related gene‐based classifier (PCSCG_ier_), *p‐*value was calculated by Pearson correlation test. (**F**) Gene set variation analysis (GSVA) showed the pathway differences between the STEM‐H and STEM‐L patients. (**G** and **H**) Systematic screening of effective small molecules for prostate cancer patients with high‐ (**G**) and low‐risk (**H**) of recurrence, respectively, *p*‐value was calculated by Student's *t*‐test.

To verify the importance of these selected PCSCGs in PCa progression and stem cell formation, we knocked down three rarely studied genes, *GINS2, FAM83D* and *C16orf59* (Figures S1C and S5.), three genes that are rarely been studied. After suppressing their expression, the PCa cell proliferation and colony formation abilities were significantly inhibited (*p* < .05, Figure 4A,B,E,F). The sphere formation ability of PCa cells was also significantly compromised (*p* < .05, Figure 4C,D). These findings supported the pivotal role of GINS2, FAM83D and C16orf59 in the sphere formation and developing of PCa, we summed the processes of this study in Figure 4G.

**FIGURE 4:**
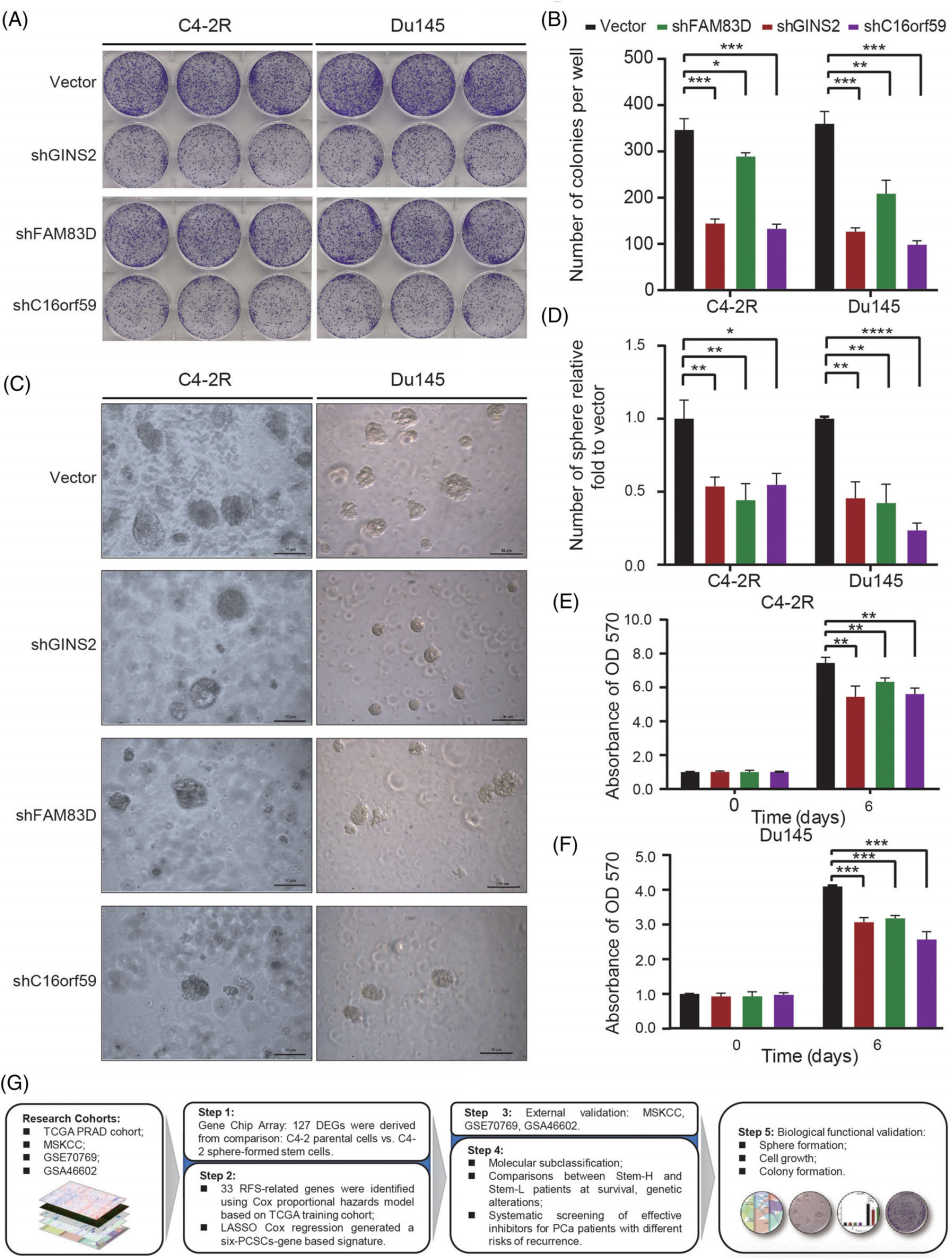
Knocking down *GINS2*, *FAM83D* and *C16orf59* significantly inhibits the proliferation, colony formation and sphere formation rate of prostate cancer cells. (**A** and **B**) Suppressing the expression of *GINS2*, *FAM83D* and *C16orf59* significantly inhibited the colony formation rates of C4‐2R and Du145 prostate cancer cells. The data were displayed with Mean ± SD; *p*‐value was calculated by Student's *t*‐test, *p ≤ .05, **p ≤ .01, ***p ≤ .001, ****p ≤ .0001. (**C** and **D**) The sphere formation difference between groups of C4‐2R and Du145 prostate cancer cell lines. The data were displayed with Mean ± SD; *p*‐value was calculated by Student's *t*‐test, *p ≤ .05, **p ≤ .01, ***p ≤ .001, ****p ≤ .0001. (**E** and **F**) Knocking down *GINS2*, *FAM83D* and *C16orf59* significantly inhibited the proliferation of prostate cancer cells. The data were displayed with Mean ± SD; *p*‐value was calculated by Student's *t*‐test, *p ≤ .05, **p ≤ .01, ***p ≤ .001, ****p ≤ .0001. (**G**) Prostate cancer stem cells (PCSCs) are a major mechanism of cancer recurrence and metastasis. We display the characteristic genes of PCSCs and based on which, a robust prognostic classifier (PCSCG_ier_) is developed. The validity and stability of PCSCG_ier_ have been proven in multiple datasets, and the function of selected critical genes has been verified. SD, standard deviation

Some biomarkers or related molecular markers differentially expressed in CSCs, which could reflect stemness and be associated with poor prognosis and the RFS of tumour.^5^, ^6^ We focused on critical CSC‐related genes, explored their biological roles in PCa and established a PCSCG_ier_ to forecast the RFS outcomes of PCa patients. We first verified its applicability and predictive value in the TCGA‐PRAD dataset and then successfully validated it in two GEO, as well the MSKCC datasets. We found that suppressing *C16orf59, GINS2 or FAM83D* expression, can significantly inhibit the proliferation, colony formation, and sphere formation abilities of PCa cells were significantly inhibited. *CDC20* ^5^, ^7^ and *FJX1* ^8^ have been reported with the stem cell‐related functions. Although the biological role of *FAM129A* in PCa has been uncovered,^9^, ^10^ few studies have been concerned with its stem cell‐related characteristics, thus future studies are warranted.

In summary, PCSCG_ier_ is a robust signature and adds prognosis prediction value for PCa patients. Prospective studies are warranted to explore its usage in clinical decision‐making and personalized treatment for PCa patients with different risks.

## CONFLICT OF INTEREST

The authors have no conflict of interest.

## Supporting information

Figure S1. Quality control. (A) First quality control showed the stem cell marker expression difference between stem cell‐enriched C4‐2 cells and regular C4‐2 cells. The data were displayed with Mean ± SD; *p*‐value was calculated by Student's *t*‐test, *p ≤ .05, **p ≤ .01, ***p ≤ .001, ****p ≤ .0001. (**B**) Second quality control showed the stem cell marker expression difference between stem cell‐enriched C4‐2 cells and regular C4‐2 cells. The data were displayed with Mean ± SD; *p*‐value was calculated by Student's t‐test, *p ≤ .05, **p ≤ .01, ***p ≤ .001, ****p ≤ .0001. (**C**) Knockdown efficiencies of GINS2, TEDC2 (also termed C16orf59) and FAM83D in Du145 and C4‐2R prostate cancer cells. SD, standard deviation
Figure S2. Nomogram receiver operating characteristic (ROC) and subgroup analyses. (A–C) Nomogram ROC synthesizes the prostate cancer stem cell‐related gene‐based classifier (PCSCG_ier_) and clinicopathological features in TCGA‐PRAD, GSE70769 and GSE46602 datasets. (**D**) Subgroup analyses based on patient age, tumour stage and Gleason score in the TCGA‐PRAD dataset, *p*‐value was calculated by log‐rank test. (**E**) Subgroup analyses based on Gleason score in the GSE70769 dataset, *p*‐value was calculated by log‐rank test.
Figure S3. Nomogram receiver operating characteristic (ROC) curve and subgroup analyses. (**A**) ROC nomogram showing the synthesis of the prostate cancer stem cell‐related gene‐based classifier (PCSCG_ier_) and clinicopathological features in the MSKCC dataset. (**B**) Subgroup analyses based on Gleason score in the MSKCC dataset, *p*‐value was calculated by log‐rank test.
Figure S4. Systematic screening of effective drugs for prostate cancer patients at low risk of recurrence. The data were displayed with Mean ± SD; *p*‐value was calculated by Student's *t*‐test, *p ≤ .05, **p ≤ .01, ***p ≤ .001, ****p ≤ .0001. SD, standard deviation
Figure S5. Uncropped and unedited blot/gel images.

Table S1. Prognostic values of differentially expressed genes between stem cell‐enriched C4‐2 and C4‐2 group.
Table S2. Clinical pathological features of recruited cohorts.
Table S3. The primer sequence of three prostate cancer stem cell‐related genes.
Table S4. Antibodies used in the current study.
