# Supplementary material for: In silico validation of a new classifier, PCSCGier, for predicting recurrence‐free survival in prostate cancer patients: Evidence from multiple datasets
Source: Clin Transl Med. 2023 Jan 15;13(1):e1105. doi: 10.1002/ctm2.1105 (PMC9841121; doi:10.1002/ctm2.1105)
Supplement: Supplementary file 1 — Figure S1. Quality control. (A) First quality control showed the stem cell marker expression difference between stem cell‐enriched C4‐2 cells and regular C4‐2 cells. The data were displayed with Mean ± SD; p‐value was calculated by Student's t‐test, *p ≤ .05, **p ≤ .01, ***p ≤ .001, ****p ≤ .0001. (B) Second quality control showed the stem cell marker expression difference between stem cell‐enriched C4‐2 cells and regular C4‐2 cells. The data were displayed with Mean ± SD; p‐value was calculated by Student's t‐test, *p ≤ .05, **p ≤ .01, ***p ≤ .001, ****p ≤ .0001. (C) Knockdown efficiencies of GINS2, TEDC2 (also termed C16orf59) and FAM83D in Du145 and C4‐2R prostate cancer cells. SD, standard deviation Figure S2. Nomogram receiver operating characteristic (ROC) and subgroup analyses. (A–C) Nomogram ROC synthesizes the prostate cancer stem cell‐related gene‐based classifier (PCSCGier) and clinicopathological features in TCGA‐PRAD, GSE70769 and GSE46602 datasets. (D) Subgroup analyses based on patient age, tumour stage and Gleason score in the TCGA‐PRAD dataset, p‐value was calculated by log‐rank test. (E) Subgroup analyses based on Gleason score in the GSE70769 dataset, p‐value was calculated by log‐rank test. Figure S3. Nomogram receiver operating characteristic (ROC) curve and subgroup analyses. (A) ROC nomogram showing the synthesis of the prostate cancer stem cell‐related gene‐based classifier (PCSCGier) and clinicopathological features in the MSKCC dataset. (B) Subgroup analyses based on Gleason score in the MSKCC dataset, p‐value was calculated by log‐rank test. Figure S4. Systematic screening of effective drugs for prostate cancer patients at low risk of recurrence. The data were displayed with Mean ± SD; p‐value was calculated by Student's t‐test, *p ≤ .05, **p ≤ .01, ***p ≤ .001, ****p ≤ .0001. SD, standard deviation Figure S5. Uncropped and unedited blot/gel images. [file CTM2-13-e1105-s001.docx]

**Fig. S1. Quality control. A.** First quality control showed the stem cell marker expression difference between stem cell-enriched C4-2 cells and regular C4-2 cells. **B.** Second quality control showed the stem cell marker expression difference between stem cell-enriched C4-2 cells and regular C4-2 cells. **C.** Knockdown efficiencies of GINS2, TEDC2 (also termed C16orf59), and FAM83D in Du145 and C4-2R prostate cancer cells.


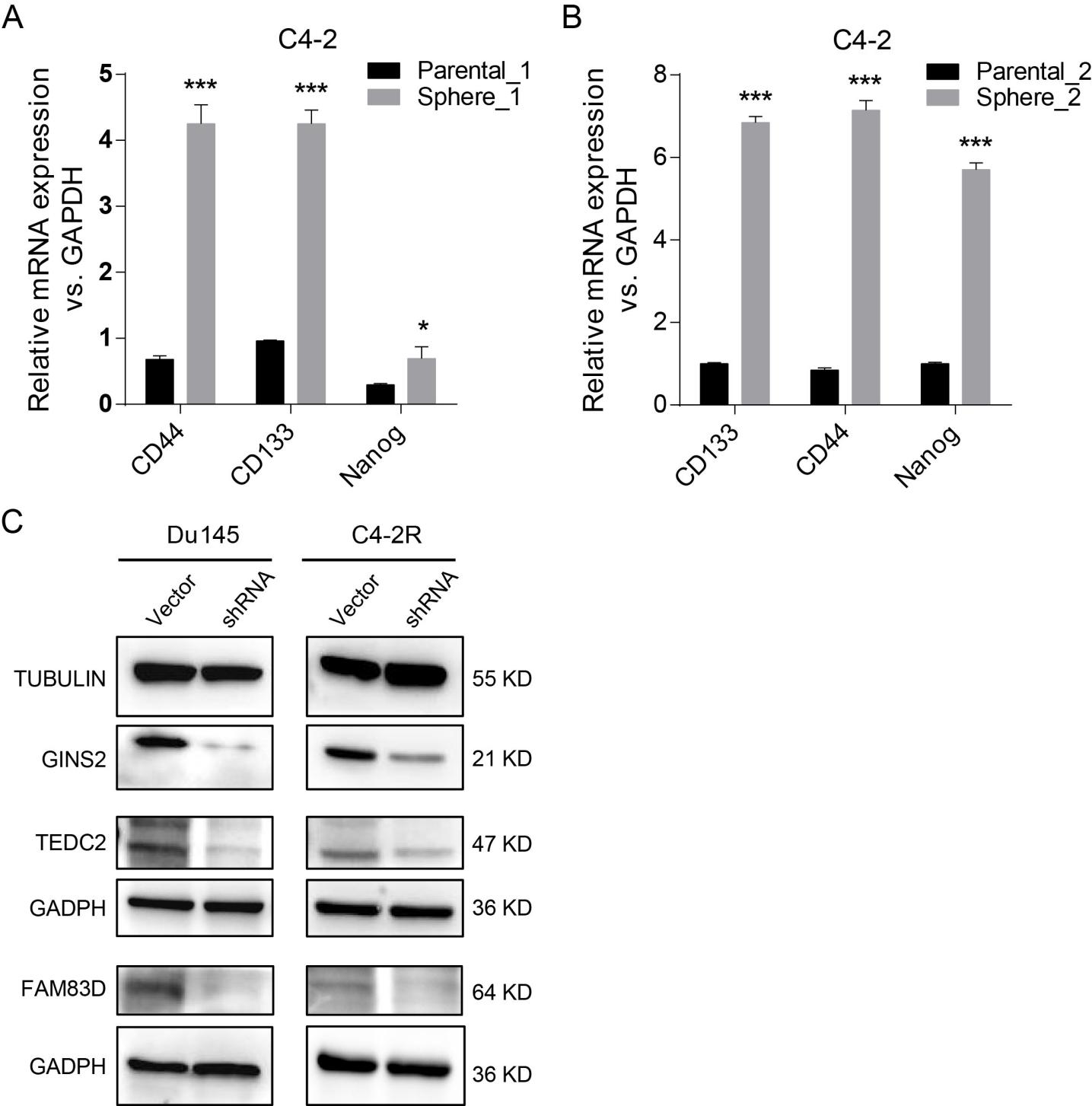


**Fig. S2. Nomogram receiver operating characteristic (ROC) and subgroup analyses. A-C.** Nomogram ROC synthesizes the prostate cancer stem cell-related gene-based classifier (PCSCG_ier_) and clinicopathological features in TCGA-PRAD, GSE70769, and GSE46602 datasets. **D.** Subgroup analyses based on patient age, tumor stage, and Gleason score in the TCGA-PRAD dataset. **E.** Subgroup analyses based on Gleason score in the GSE70769 dataset.


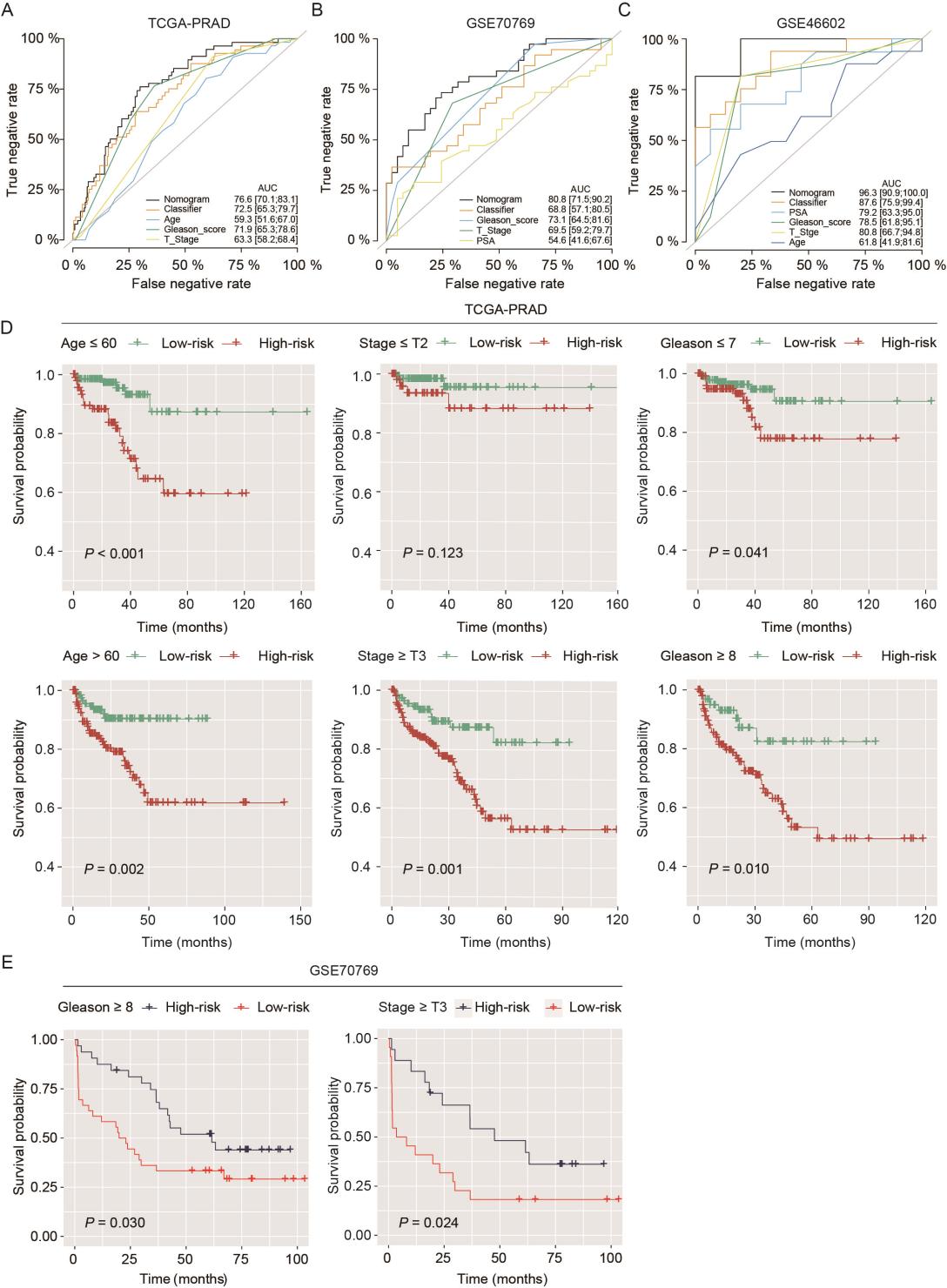


**Fig. S3. Nomogram receiver operating characteristic (ROC) curve and subgroup analyses. A.** ROC nomogram showing the synthesis of the prostate cancer stem cell-related gene-based classifier (PCSCG_ier_) and clinicopathological features in the MSKCC dataset. **B.** Subgroup analyses based on Gleason score in the MSKCC dataset.


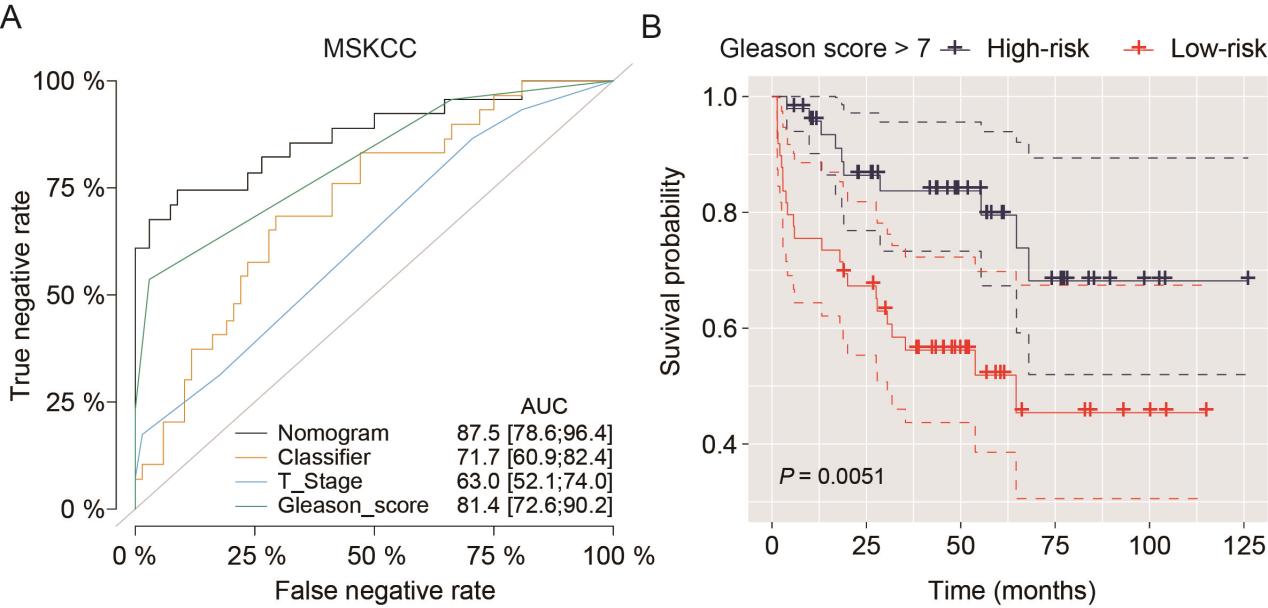


**Fig. S4. Systematic screening of effective drugs for prostate cancer patients at low risk of recurrence.**

**
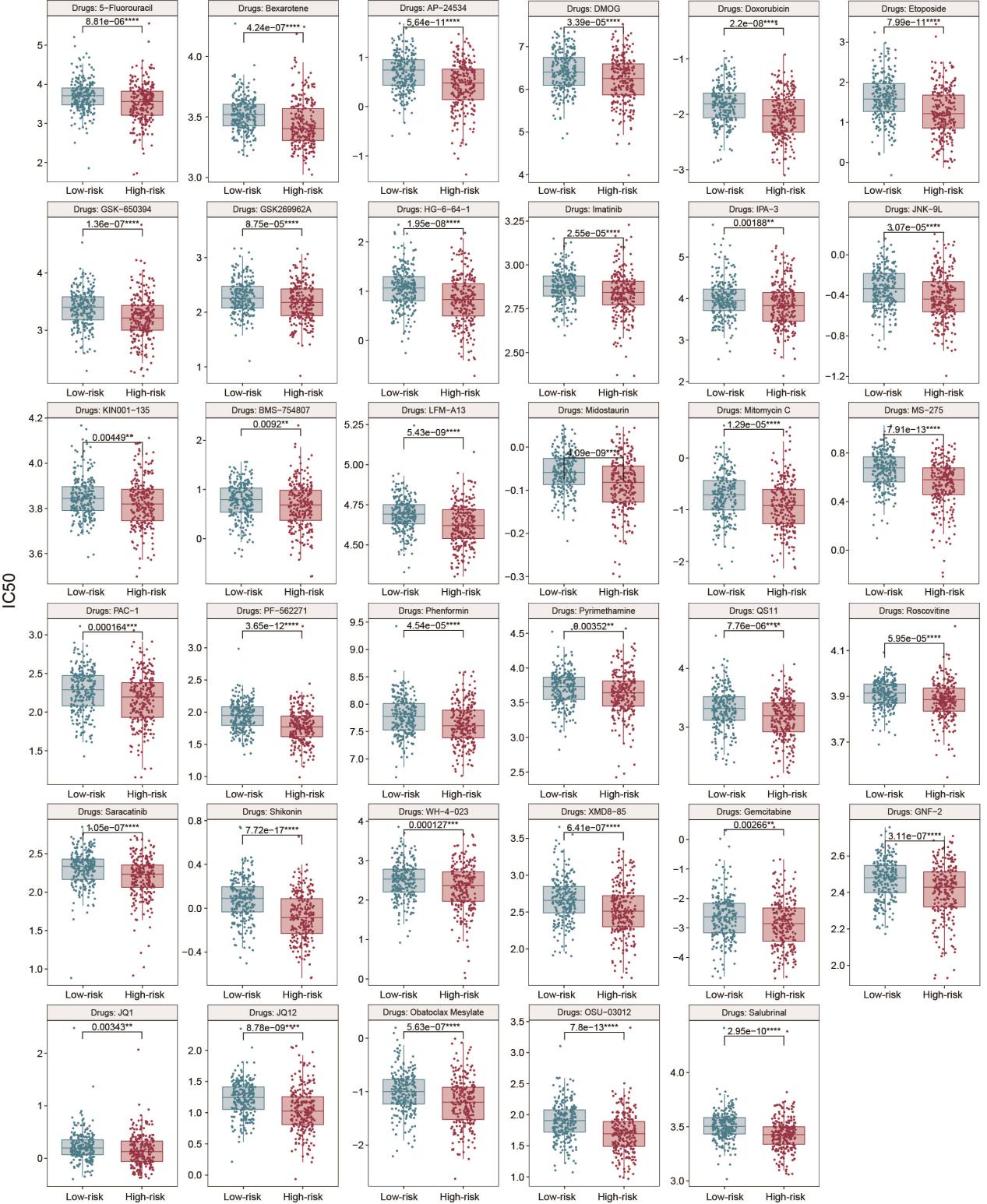
**

**Fig. S5. Uncropped and unedited blot/gel images.**


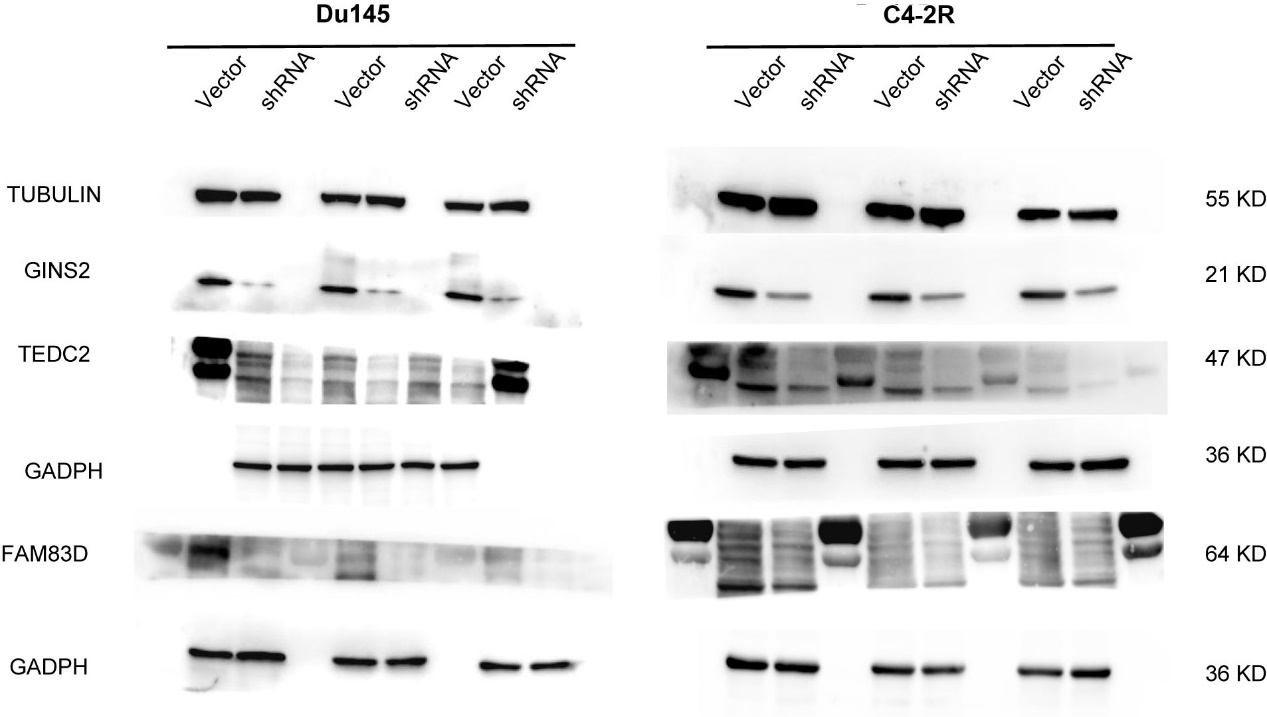


**Table S1. Prognostic values of differentially expressed genes between stem cell-enriched C4-2 and C4-2 group.**

| **Gene symbol** | **logFC** | **Effect size** | ***P*-value** | **β-value** | **HR** | **HR.95L** | **HR.95H** | **Cox *P*-value** | **Log-rank *P*-value** | **LASSO Cox Co-efficient** |
| --- | --- | --- | --- | --- | --- | --- | --- | --- | --- | --- |
| AADAT | 3.034182652 | -381.7391361 | 0.000204645 | 0.0009988 | 1.000999341 | 0.966599879 | 1.036623015 | 0.955355411 | 0.955405219 | / |
| ABCC3 | -3.062181726 | 16.44847224 | 0.028809091 | 0.0332700 | 1.033829757 | 0.882918006 | 1.210535926 | 0.679422295 | 0.679267515 | / |
| AIM1 | 3.10133845 | 27.19446496 | 0.017284284 | -0.008115 | 0.99191831 | 0.973895469 | 1.010274681 | 0.385757173 | 0.387058662 | / |
| AKR1C3 | -4.432707309 | -116.0871231 | 0.003990998 | 0.0436500 | 1.044614104 | 1.00214512 | 1.088882843 | 0.039288369 | 0.032102587 | / |
| ANO7 | 3.638539881 | 17.16615346 | 0.027635673 | -0.015160 | 0.984952856 | 0.973404642 | 0.996638074 | 0.011748667 | 0.012121607 | / |
| AURKB | 4.661145731 | 217.9232655 | 0.000370101 | 0.1411000 | 1.151495692 | 1.023444696 | 1.295568128 | 0.019014353 | 0.016702713 | / |
| C16orf59 | 3.118733335 | 95.28045019 | 0.002490698 | 0.3341000 | 1.396679638 | 1.150626749 | 1.69534909 | 0.000727501 | 0.000641222 | 0.014613847 |
| C5 | 3.644640526 | 57.49639729 | 0.00768825 | -0.067510 | 0.934717302 | 0.80648855 | 1.083333961 | 0.369852418 | 0.366888119 | / |
| CCNA2 | 3.271037431 | 416.8900019 | 0.000133655 | 0.1051000 | 1.110786562 | 1.02379181 | 1.205173527 | 0.011568457 | 0.010239629 | / |
| CDC20 | 3.13992454 | 207.2176337 | 0.000781363 | 0.0894300 | 1.093554983 | 1.049915922 | 1.139007872 | 1.68E-05 | 6.06E-06 | 0.027988086 |
| CDC6 | 3.861230268 | 67.15424695 | 0.003406965 | 0.1107000 | 1.117074027 | 0.939052237 | 1.328844481 | 0.211306112 | 0.212412228 | / |
| CDCA5 | 4.364535516 | 25.4915663 | 0.018366599 | 0.1271000 | 1.135486484 | 1.050118026 | 1.227794898 | 0.001441144 | 0.000914219 | / |
| CDCA7 | 3.058913801 | 105.0783686 | 0.001300104 | 0.1334000 | 1.142703281 | 0.964018018 | 1.354508695 | 0.124151941 | 0.123831617 | / |
| CDCA7L | 3.056717458 | 54.03567332 | 0.004639623 | 0.0303500 | 1.030820251 | 0.927953747 | 1.145089821 | 0.571446658 | 0.571161034 | / |
| CDH26 | 3.009212662 | 103.3837761 | 0.001359527 | -0.018140 | 0.982025372 | 0.909362054 | 1.060494911 | 0.64376064 | 0.643921603 | / |
| CDT1 | 3.345892431 | 15.65918735 | 0.027378644 | 0.0862300 | 1.090058124 | 1.013695152 | 1.172173618 | 0.019963449 | 0.016767273 | / |
| CENPU | 3.164132218 | 67.37407221 | 0.002933444 | 0.0924100 | 1.096818512 | 1.008879113 | 1.192423187 | 0.03021374 | 0.028387309 | / |
| CENPW | 3.32996487 | 283.2071312 | 0.000352648 | 0.1124000 | 1.118944079 | 1.034935111 | 1.209772321 | 0.004768022 | 0.004566072 | / |
| DSCC1 | 3.609752338 | 162.4313679 | 0.000631571 | 0.2380000 | 1.268700952 | 1.078772002 | 1.492068854 | 0.004022986 | 0.003809622 | / |
| DUSP5 | -3.243937951 | -36.94555824 | 0.008931205 | -0.003737 | 0.996270279 | 0.97593645 | 1.017027767 | 0.722470058 | 0.722579386 | / |
| ERICH2 | -4.049984654 | -47.96007954 | 0.00849121 | 0.065430 | 1.067616624 | 0.959477535 | 1.187943662 | 0.229834699 | 0.233384773 | / |
| ETV5 | -3.625266228 | -4983.137813 | 5.13E-05 | -0.009909 | 0.990140008 | 0.949702176 | 1.032299663 | 0.641388852 | 0.569763304 | / |
| FAM129A | -3.110956408 | 22.8396855 | 0.014721926 | -0.026680 | 0.973668312 | 0.956651345 | 0.990987976 | 0.003014118 | 0.002924985 | -0.009740136 |
| FAM83D | 3.494585407 | 28.12695991 | 0.012326184 | 0.0704600 | 1.072999058 | 1.042618587 | 1.104264775 | 1.52E-06 | 6.90E-07 | 0.046377822 |
| FBXO5 | 3.48795133 | 1123.299491 | 0.002502267 | 0.1362000 | 1.145862716 | 0.824386971 | 1.592700287 | 0.417672738 | 0.419528892 | / |
| FEN1 | 4.677635962 | 447.3346032 | 0.000400041 | 0.0643000 | 1.066416945 | 1.016624711 | 1.118647902 | 0.008393949 | 0.009639678 | / |
| FERMT1 | 3.206841784 | 18.99320471 | 0.020752676 | -0.150200 | 0.860558608 | 0.744457086 | 0.994766698 | 0.042262684 | 0.042476542 | / |
| FEZ1 | -3.276492238 | 12.97403083 | 0.036551637 | 0.0143900 | 1.014496045 | 0.784610216 | 1.311736968 | 0.912587942 | 0.912536704 | / |
| FJX1 | 3.026959523 | 48.97695459 | 0.007569131 | 0.2355000 | 1.265543094 | 1.082879015 | 1.479019631 | 0.003065199 | 0.003030044 | 0.037413058 |
| GINS2 | 3.666425881 | 38.12932285 | 0.011419715 | 0.1717000 | 1.187305938 | 1.081390724 | 1.303594861 | 0.00031665 | 0.000395054 | 0.05030616 |
| GRPR | 3.819639837 | 36.09039909 | 0.009791015 | -0.014000 | 0.986102205 | 0.894938028 | 1.086552955 | 0.777353327 | 0.777588418 | / |
| GUCY1A3 | 3.198065735 | -15.91819765 | 0.028404775 | -0.005181 | 0.994832725 | 0.979793394 | 1.010102902 | 0.505040614 | 0.505186059 | / |
| HIST1H2AM | 3.187279665 | 15.47467183 | 0.030138313 | 0.0160500 | 1.016175583 | 0.896007841 | 1.152459575 | 0.802668375 | 0.80247271 | / |
| HIST1H3E | 4.030859742 | 145.5865679 | 0.002779669 | -0.021190 | 0.979030103 | 0.923346096 | 1.038072231 | 0.478117922 | 0.478147651 | / |
| HIST2H2AC | 3.671679375 | -212.1897779 | 0.000432721 | 0.0037690 | 1.003776449 | 0.90920374 | 1.108186333 | 0.940487231 | 0.940417387 | / |
| HMMR | 3.541163672 | 50.31903471 | 0.004297209 | 0.1848000 | 1.202985016 | 1.070079257 | 1.352397908 | 0.001975435 | 0.001652889 | / |
| HSPA6 | -5.156834699 | 29.34261498 | 0.015706566 | -0.007459 | 0.99256918 | 0.955029211 | 1.031584758 | 0.704567352 | 0.703052745 | / |
| IFIT1 | -3.231004149 | -66.47236398 | 0.004336365 | 0.031440 | 1.031940187 | 1.004939867 | 1.05966594 | 0.02011248 | 0.017356662 | / |
| IFIT2 | -3.606351209 | 11.9222683 | 0.039334551 | 0.037100 | 1.037795641 | 0.989582158 | 1.088358134 | 0.126390336 | 0.119596263 | / |
| IFIT3 | -3.929773353 | 9.652495822 | 0.048781132 | 0.022870 | 1.023129974 | 0.99520637 | 1.051837061 | 0.105314252 | 0.100915389 | / |
| KIAA0101 | 4.319946186 | 68.72026064 | 0.003602325 | 0.148000 | 1.159546735 | 1.021342744 | 1.316451934 | 0.022248124 | 0.020388706 | / |
| KIAA1683 | -3.397992004 | 130.427649 | 0.001704963 | 0.072840 | 1.075554185 | 0.948498544 | 1.219629501 | 0.256128878 | 0.249878556 | / |
| KIF11 | 3.720071946 | 650.6503855 | 0.000271556 | 0.138400 | 1.148418379 | 1.035411553 | 1.27375899 | 0.008834431 | 0.007717423 | / |
| KIF20A | 3.885985564 | 531.6038775 | 8.08E-05 | 0.217100 | 1.242431477 | 1.118895977 | 1.379606332 | 4.86E-05 | 2.88E-05 | / |
| KIF4A | 4.056297946 | 36.36644421 | 0.007732578 | 0.113100 | 1.119714142 | 1.043258896 | 1.201772412 | 0.001726849 | 0.001001584 | / |
| MCM3 | 3.491370517 | 46.35922167 | 0.009287247 | 0.038700 | 1.03945943 | 0.996070461 | 1.084738429 | 0.075243494 | 0.076349909 | / |
| MCM4 | 3.90292846 | 79.06154272 | 0.005828688 | 0.056330 | 1.057949612 | 0.999192107 | 1.120162353 | 0.05332969 | 0.05063745 | / |
| MCM5 | 3.444888837 | 96.219071 | 0.001683964 | 0.166100 | 1.180641698 | 1.018862322 | 1.368109105 | 0.027210377 | 0.027297494 | / |
| MKI67 | 3.762551208 | 29.59444402 | 0.015763592 | 0.152000 | 1.164109345 | 1.064279086 | 1.273303765 | 0.000894359 | 0.000647858 | / |
| MYBL2 | 3.086410305 | 61.01520294 | 0.007578476 | 0.043900 | 1.04488065 | 1.020014882 | 1.070352591 | 0.000353456 | 0.000138356 | / |
| PBK | 3.614000834 | 101.9840455 | 0.00310408 | 0.112900 | 1.119486576 | 0.996752262 | 1.257333685 | 0.056772477 | 0.053698865 | / |
| PLK2 | -3.709612641 | 9.553780577 | 0.049858423 | 0.046990 | 1.048111446 | 0.965984033 | 1.137221284 | 0.259029536 | 0.260513734 | / |
| RAD51AP1 | 3.886893738 | -735.804198 | 0.000127036 | 0.087830 | 1.09180779 | 0.87521055 | 1.362008548 | 0.43625612 | 0.436973434 | / |
| SLC26A3 | -3.142936527 | -34.49867569 | 0.007812247 | -0.0004566 | 0.999543479 | 0.97631395 | 1.023325711 | 0.969639557 | 0.969670762 | / |
| SLC4A4 | 3.820288523 | 25.05694403 | 0.016742119 | -0.007003 | 0.993021833 | 0.986173107 | 0.999918121 | 0.047350317 | 0.046274016 | / |
| SNCA | -3.572683411 | 13.63961302 | 0.034966143 | 0.026670 | 1.027032535 | 0.988726251 | 1.066822922 | 0.169020109 | 0.16575235 | / |
| SPC24 | 4.134058328 | -101.6546548 | 0.002678126 | 0.185500 | 1.203761712 | 1.076213661 | 1.346426191 | 0.001173391 | 0.001047553 | / |
| STAG1 | 3.158970905 | -49.28967928 | 0.008386588 | -0.003744 | 0.996262524 | 0.856950236 | 1.158222467 | 0.961140842 | 0.961199293 | / |
| TACC3 | 3.288288934 | 891.0039823 | 5.75E-05 | 0.126200 | 1.13452633 | 1.053497566 | 1.221787344 | 0.000842491 | 0.000589789 | / |
| TMEFF2 | 3.616743781 | 33.04260813 | 0.008634186 | -0.001518 | 0.998483279 | 0.994491135 | 1.00249145 | 0.457730999 | 0.457908814 | / |
| TONSL | 3.140400647 | -840.6792146 | 6.98E-05 | 0.221900 | 1.2485062 | 1.095986899 | 1.422250333 | 0.00084167 | 0.001113064 | / |
| TOP2A | 3.820288645 | 268.2120278 | 0.000331247 | 0.053730 | 1.055203136 | 1.010101186 | 1.10231893 | 0.015912714 | 0.014379878 | / |
| TRIP13 | 3.454958329 | 21.23321842 | 0.021942505 | 0.204900 | 1.227368059 | 1.054650507 | 1.428371145 | 0.008106556 | 0.00732847 | / |
| TUBA1B | 3.450535325 | 632.1743151 | 0.00012539 | 0.017640 | 1.01779146 | 0.999400217 | 1.036521143 | 0.058029131 | 0.062363148 | / |
| TYMS | 3.310528296 | 11.64045173 | 0.041164362 | 0.078540 | 1.081701586 | 1.017345686 | 1.150128553 | 0.012091456 | 0.010974744 | / |
| ZWINT | 4.573992379 | 235.5198247 | 0.000512942 | 0.040380 | 1.041204562 | 0.996048808 | 1.088407447 | 0.074269012 | 0.075526422 | / |

logFC, log (Fold change); HR, hazard ratio; 95L, Low 95%CI; 95H, High 95%CI.

**Table S2. Clinical pathological features of recruited cohorts.**

| **Parameters** | **TCGA-PRAD** | **MSKCC** | **GSE70769** | **GSE46602** |
| --- | --- | --- | --- | --- |
| Number | 495 | 140 | 90 | 34 |
| Age (Mean ± SD) | 61.03±6.83 | - | - | 62.44±5.63 |
| Gleason score |  |  |  |  |
| < 7 | 45 | 41 | 18 | 16 |
| 7 | 246 | 76 | 53 | 14 |
| > 7 | 204 | 21 | 14 | 4 |
| Tumor Stage |  |  |  |  |
| ≤ T2 | 187 | 133 | 47 | 18 |
| ≥ T3 | 301 | 6 | 38 | 16 |

**Note:** TCGA-PRAD, The Cancer Genome Atlas-Prostate adenocarcinoma; MSKCC, memorial sloan-kettering cancer center; SD, Standard Deviation.

**Table S3. The shRNA primer sequence of three prostate cancer stem cell-related genes.**

| **ID** | **Gene symbol** | **Forward primer** | **Reverse primer** |
| --- | --- | --- | --- |
| 1 | C16orf59 | GTTCGAAGAGGCATCACTAAG | CTTAGTGATGCCTCTTCGAAC |
| 2 | GINS2 | GAATGGATTCAGGATGTTGTT | AACAACATCCTGAATCCATTC |
| 3 | FAM83D | CAGTTCGGACTATCACAGGAA | TTCCTGTGATAGTCCGAACTG |

**Table S4. Antibodies used in the current study.**

| **Antibodies** | **Source** | **Identifier** |
| --- | --- | --- |
| GINS2 Antibody | Affinity Biosciences | Cat# DF9451 |
| FAM83D Antibody | Bioss Antibodies | Cat# bs-14678R |
| C16orf59 Antibody | Novus Biologicals | Cat# NBP2-85897 |
| β-tubulin Antibody | Affinity Biosciences. | Cat# T0023 |
| GAPDH Antibody | Affinity Biosciences LTD. | Cat# AF7021 |
| Goat Anti-Rabbit IgG | Elabscience | Cat# E-AB-1003 |
| Goat Anti-Mouse IgG | Elabscience | Cat# E-AB-1001 |
