# Supplementary material for: In silico validation of a new classifier, PCSCGier, for predicting recurrence‐free survival in prostate cancer patients: Evidence from multiple datasets
Source: Clin Transl Med. 2023 Jan 15;13(1):e1105. doi: 10.1002/ctm2.1105 (PMC9841121; doi:10.1002/ctm2.1105)
Supplement: Supplementary file 2 — Table S1. Prognostic values of differentially expressed genes between stem cell‐enriched C4‐2 and C4‐2 group. Table S2. Clinical pathological features of recruited cohorts. Table S3. The primer sequence of three prostate cancer stem cell‐related genes. Table S4. Antibodies used in the current study. [file CTM2-13-e1105-s002.docx]

**Letter-to-Editor**

***In silico* validation of a new classifier, PCSCG_ier_, for predicting** **recurrence-free survival in prostate cancer patients:** *Evidence from multiple datasets*

**Running Head: PCSCG_ier_, a classifier for predicting RFS of PCa**

Meng Zhang^1,2,#^, Zichen Bian^1,#^, Jia Chen^1^, Lei Chen^1^, Jun Zhou^1^, Qingsong Niu^1^, Zongyao Hao^1^, Jialin Meng^1,2,^*, Chaozhao Liang^1,^*

^1^Department of Urology, The First Affiliated Hospital of Anhui Medical University, Hefei, China; Institute of Urology, Anhui Medical University, Hefei, China; Anhui Province Key Laboratory of Genitourinary Diseases, Anhui Medical University, Hefei, China.

^2^Institute of Urology of Shenzhen University, The Third Affiliated Hospital of Shenzhen University, Shenzhen Luohu Hospital Group, Shenzhen 518000, China.

^#^These authors contributed equally to the work.

***Correspondence to**

Jialin Meng ([mengjialin@ahmu.edu.cn](mailto:mengjialin@ahmu.edu.cn))

**Affiliation:** ^1^Department of Urology, The First Affiliated Hospital of Anhui Medical University, Hefei, China; Institute of Urology, Anhui Medical University, Hefei, China; Anhui Province Key Laboratory of Genitourinary Diseases, Anhui Medical University, Hefei, China.

**Tel.:** +86 55162922234, and Fax.: +86 55162922034

**Address:** Jixi Road 218, Shushan District, Hefei City 230022, Anhui Province, People’s Republic of China.

Chaozhao Liang ([liang_chaozhao@ahmu.edu.cn](mailto:liang_chaozhao@ahmu.edu.cn)).

**Affiliation:** ^1^Department of Urology, The First Affiliated Hospital of Anhui Medical University, Hefei, China; Institute of Urology, Anhui Medical University, Hefei, China; Anhui Province Key Laboratory of Genitourinary Diseases, Anhui Medical University, Hefei, China.

**Tel.:** +86 55162922234, and Fax.: +86 55162922034

**Address:** Jixi Road 218, Shushan District, Hefei City 230022, Anhui Province, People’s Republic of China.

**Materials and Methods**

*Cell culture*

C4-2 and Du145 cells were purchased from the American Type Culture Collection (Manassas, VA, USA). All these cells were cultured in Roswell Park Memorial Institute (RPMI) 1640 (Gibco™, Inc., USA) medium containing 10% fetal bovine (Gibco™, Inc., USA) serum plus 1% penicillin and streptomycin (Gibco™, Inc., USA) at 100 U/ml and 100 mcg/ml, respectively, at 37°C and 5% CO_2_. The C4-2R cells were developed by maintaining with 10 µM enzalutamide (MedChemExpress, Monmouth Junction, NJ, USA) for at least six months[1, 2]. After long-term drug selection, PCa cells were no longer sensitive to enzalutamide treatment and became enzalutamide-resistant cells.

*Sphere formation, RNA extraction, Gene Chip profiling, and bioinformatic analyses*

Before starting the experiment, growth factor-reduced Matrigel™ was thawed on ice (usually 4-5 hours). The C4-2 cells were digested and counted to make single-cell suspensions. To generate spheres from this PCa cell line, we plated 10,000 cells/well in three 24-well plates. The cold cell suspension was mixed with cold Matrigel™ at a ratio of 1:1 (50 μL cell suspension:50 μL Matrigel™). Then, 100 μL of the mixture was slowly pipetted into the bottom rim of each well of a 24-well plate. The plate was placed in an incubator at 37°C for 1 hour. After that, 1 mL of warm serum-free medium (RPMI) was transferred into each well without disturbing the gels (termed sphere cells). The whole procedure of sphere formation took 10-14 days.

Then, we used the cell recovery solution to dissolve the Matrigel to obtain purified stem cells. Total RNA was extracted from them, and the quality and integrity of the total RNA were assessed by an Agilent 2100 bioanalyzer and RNA LabChip kits (Agilent Technologies Inc., Santa Clara, CA, USA). We tested the quality of these stem cell-enriched RNAs by measuring classic stem cell markers, including CD44, CD133, and Nanog (**Fig. S1A-B**). The sequencing (Agilent LncRNA Chip, Agilent Technologies Inc., Santa Clara, CA, USA) and analytical procedures were performed by Shanghai OE Biotech. Co., Ltd. (Shanghai, China). Genes whose adjusted *P* value was < 0.05 and absolute [log_2_ (fold change)] was > 1.0 were defined as DEGs[3-5]. These DEGs were termed PCSC-related genes (PCSCGs).

*Construction and validation of the PCSCG-based classifier*

This study used the data of patients with PCa from four datasets, the Cancer Genome Atlas (TCGA), Memorial Sloan-Kettering Cancer Center (MSKCC), GSE70769, and GSE46602. A summary of the characteristics of each dataset is provided in **Table S2**. For the TCGA-PRAD dataset, we obtained the transcripts per kilobase million (TPM) values via “TCGAbiolinks” and then transformed them to log_2_(TPM+1) values for downstream analysis. For the MSKCC dataset, the normalized log_2_(mRNA) expression was downloaded from <http://cbio.mskcc.org/cancergenomics/prostate/data/>. The gene matrix data of GSE70769 and GSE46602 were downloaded from the Gene Expression Omnibus (GEO), and the expression value of each gene was pre-log_2_-transformed.

We selected the PCSCGs with a false discovery rate (FDR) < 0.05 and absolute log_2_-fold change in gene expression > 3. The TCGA dataset was used as the training set. We first identified RFS-related PCSCGs by univariate Cox regression analysis, and then LASSO Cox regression analysis was done to construct the PCSC-related gene-based classifier (PCSCG_ier_) based on the expression of the PCSCGs and their matched coefficients calculated by *the* “survival” and “glmnet” R packages. The risk score of each patient based on PCSCG_ier_ was calculated as: risk score =
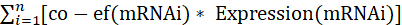
. The median value of the risk score in each dataset was used as the cutoff value to separate patients into low-risk and high-risk subgroups. With the formula mentioned above, we calculated the risk score of each patient from the MSKCC dataset, GSE70769 dataset, and GSE46602 dataset. The median value of the risk score in each dataset was applied to divide patients into low- and high-risk subgroups.

*Pathway enrichment, genetic alteration, and prediction of therapeutic drugs*

Gene set enrichment analysis (GSEA, http://www.broadinstitute.org/gsea/index.jsp) was performed to determine which gene sets and pathways were enriched in the high-risk vs. The low-risk group. Copy number alteration (CNA) data were generated by GISTIC2.0 from GDAC Firehose (https://gdac.broadinstitute.org). We compared amplification and deletion events at both the focal and arm levels between the high-risk and low-risk groups. The mutation data were retrieved from TCGA (https://tcga-data.nci.nih.gov), and we calculated the number of nonsynonymous mutations per million bases to evaluate the tumor mutation burden (TMB). Using the “GSVA” R package, we performed gene set variation analysis (GSVA) to assess the variations in the pathway activities between patients belonging to the high- and low-risk groups based on 50 hallmark genes. Drug sensitivity and phenotype data from GDSC 2016 (https://www.cancerrxgene.org/) were used to predict the chemotherapeutic response to three drugs via the R package “MOVICS” [6].

*shRNA delivery and function assay*

We obtained sh*GINS2*, sh*FAM83D,* and sh*C16orf59* lentiviruses from Sangon Biotech (Sangon Biotech Co., Ltd., Shanghai, China) at a concentration of 1 × 10^8^ TU/ml. Briefly, sh*GINS2*, sh*FAM83D,* and sh*C16orf59* were inserted individually into the PDS126_pL-U6-shRNA-GFP vector. The shRNA sequences for each gene are listed in **Table S3**. The same amount of virus was added to the vector and shRNA plates to obtain stably transfected cell lines. The knockdown efficiencies were determined by western blotting assay, and the detailed procedures were referring to our recent studies[1, 2, 7]. The antibodies used in the current study are summarized in **Table S4**.

The 3-(4,5-dimethylthiazol-2-yl)-2,5-diphenyltetrazoliumbromide (MTT), colony formation, and sphere formation (**see above**) assays were used to assess PCa cell function after knocking down the expression of the *GINS2, FAM83D and C16orf59* genes (**Fig. S1C**). Briefly, each well of a 24-well plate was seeded with 5000 cells in 500 µL medium. After six days of culture, 50 µL MTT in prepped 5 mg/mL MTT reagent was added to each well. The cells were incubated at 37°C for 1.5 hours. Then, dimethyl sulfoxide (DMSO) was used to dissolve the formazan. Absorbance was measured at 570 nm (Infinite 200 PRO, Tecan, Grodlg, Austria). For the colony formation assay, C4-2R and Du145 cells were seeded in 6-well plates at a density of 1000 cells/well. The cells were cultured for 14 days. After that, 4% paraformaldehyde was used to fix the cells on the plate for 20 minutes, and the cells were stained with 0.05% crystal violet for 20 minutes. After capture, we employed ImageJ software to quantify the colony formation rate.

*Compare PCSCG_ier_ with proposed molecular subtypes*

We previously classified the TCGA-PRAD patients into non-immune, immune-activated, and immune-suppressed subtypes by the non-negative matrix factorization[8]. Thorsson et al. [9] also reported six pan-cancer immune subtypes. In the current study, we explored the association between our PCSCG_ier_ subgroups and the above-mentioned two immune subtypes.

*Statistical analyses*

Multiline receiver operating characteristic (ROC) curves (nomogram included) were drawn to test the independence of the signature and compare its predictive value with that of other clinicopathological features. We also evaluated the application value of this model in predicting the RFS of PCa patients with different clinicopathological features, such as age, Gleason score, and tumor stage. Gene set enrichment analysis (GSEA) (http://www.broadinstitute.org/gsea/index.jsp) was performed to determine gene sets and pathways enriched in the high-risk vs. the low-risk group, along with the biological processes from the Kyoto Encyclopedia of Genes and Genomes (KEGG) pathways[10].

We compared the clinical RFS outcomes of subgroups by Kaplan–Meier and log-rank analysis and performed ROC curve analysis to evaluate the prognostic accuracy of PCSCG_ier_ with the “survival” and “pROC” R packages. To compare the continuous data between groups, Student’s t test or the Wilcoxon rank-sum test was selected, depending on the normality of the data. All analyses were run with R software version 4.1.0. A *P* value less than 0.05 was considered statistically significant.

**Reference:**

[1] Zhang M, Sun Y, Huang CP, Luo J, Zhang L, Meng J, et al. Targeting the Lnc-OPHN1-5/androgen receptor/hnRNPA1 complex increases Enzalutamide sensitivity to better suppress prostate cancer progression. Cell Death Dis. 2021;12(10):855.

[2] Zhang M, Sun Y, Meng J, Zhang L, Liang C, Chang C. Targeting AR-Beclin 1 complex-modulated growth factor signaling increases the antiandrogen Enzalutamide sensitivity to better suppress the castration-resistant prostate cancer growth. Cancer Lett. 2019;442:483-90.

[3] Wen J, Liu Y, Zhan Z, Chen S, Hu B, Ge J, et al. Comprehensive analysis of mRNAs, lncRNAs and circRNAs in the early phase of microglial activation. Exp Ther Med. 2021;22(6):1460-.

[4] Hou M, Zhang Y, Zhou X, Liu T, Yang H, Chen X, et al. Kartogenin prevents cartilage degradation and alleviates osteoarthritis progression in mice via the miR-146a/NRF2 axis. Cell Death Dis. 2021;12(5):483-.

[5] Zhu H, Li J, Li Y, Zheng Z, Guan H, Wang H, et al. Glucocorticoid counteracts cellular mechanoresponses by LINC01569-dependent glucocorticoid receptor-mediated mRNA decay. Sci Adv. 2021;7(9):eabd9923.

[6] Lu X, Meng J, Zhou Y, Jiang L, Yan F. MOVICS: an R package for multi-omics integration and visualization in cancer subtyping. Bioinformatics. 2020.

[7] Meng J, Guan Y, Wang B, Chen L, Chen J, Zhang M, et al. Risk subtyping and prognostic assessment of prostate cancer based on consensus genes. Commun Biol. 2022;5(1):233-.

[8] Meng J, Zhou Y, Lu X, Bian Z, Chen Y, Zhou J, et al. Immune response drives outcomes in prostate cancer: implications for immunotherapy. Mol Oncol. 2021;15(5):1358-75.

[9] Thorsson V, Gibbs DL, Brown SD, Wolf D, Bortone DS, Ou Yang TH, et al. The Immune Landscape of Cancer. Immunity. 2018.

[10] Kanehisa M, Furumichi M, Tanabe M, Sato Y, Morishima K. KEGG: new perspectives on genomes, pathways, diseases and drugs. Nucleic Acids Res. 2017;45(D1):D353-D61.
